# Trapping the intruder — immune receptor domain fusions provide new molecular leads for improving disease resistance in plants

**DOI:** 10.1186/s13059-016-0891-6

**Published:** 2016-02-19

**Authors:** Simon G. Krattinger, Beat Keller

**Affiliations:** Department of Plant and Microbial Biology, University of Zürich, Zollikerstrasse 107, 8008 Zürich, Switzerland

## Abstract

A new study uses genomics to show that fusions of plant immune receptors and other protein domains occur in significant numbers. This finding will generate many new research hypotheses and provide new opportunities for breeding resistant plant varieties.

Please see related Research article: http://dx.doi.org/10.1186/s12915-016-0228-7

Plants are constantly challenged by potential and actual pathogenic microbes, including viruses, bacteria and fungi, and so have evolved sophisticated molecular defense mechanisms that recognize, stop or slow down potential pathogens. Damage to host plants caused by microbial organisms is of particular importance in agriculture where plant diseases are a major cause of crop losses. Upon infection, microbes inject into host cells a set of virulence effectors tailored to target protein domains that are involved in the host plant’s basal immunity, thus disarming or disabling the plant’s immune response. When fused to intracellular immune receptors, however, the same protein domains become ‘baits’ that plants use to detect effector molecules and to activate defense responses. In a recent article in *BMC Biology*, Sarris et al. [1] expand our knowledge of this neat plant defense mechanism by highlighting the large number and diversity of such chimeric immune receptors.

## Intracellular receptors play a key role in the plant immune system

The immune system of plants is composed of two tiers of receptors [2]. The first tier confers basal resistance to the majority of ‘would-be’ pathogens. Basal immunity is conferred by receptors at the external face of the host cell that recognize conserved microbial structures such as the flagella of bacteria or the fungal cell wall component chitin. Microbes need to disarm this first layer of defense with a matching set of virulence effectors in order to successfully invade a host cell. For example, the RIN4 protein of *Arabidopsis* is a master regulator of basal defense and it is the target of several bacterial effector proteins [3]. The second tier of defense receptors consists of intracellular receptors belonging to the conserved family of nucleotide-binding, leucine-rich repeat proteins (NLRs), which recognize specific effector molecules. The NLR-based defense response is much stronger than basal resistance and often results in the death of the affected host cell.

**Fig. 1:**
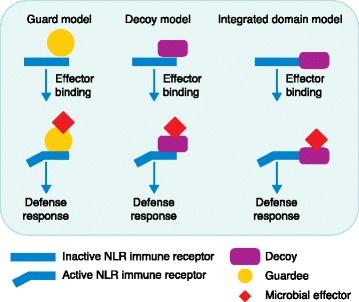
Comparison of different effector recognition models by intracellular plant nucleotide-binding, leucine-rich repeat (*NLR*) immune receptors. In the guard model, NLR receptors perceive modifications in guardees that are introduced by microbial effector proteins. Guardees often have a function in basal plant defense and they are therefore frequently targeted by effectors. The decoy is a duplicated guardee without a function in plant immunity. Its sole role is to trap effectors, thereby activating the immune signaling cascade. The guardee or decoy and the monitoring NLR are often encoded by different genes that likely bind to each other, but can dissociate. The integrated domain model involves a fusion between the decoy domain and the respective NLR, which together are encoded by one gene

Effector perception by NLR immune receptors occurs either directly or indirectly. Indirect recognition can be achieved when NLR receptors monitor the activity of effectors on other host proteins involved in basal plant immunity. This is referred to as the ‘guard’ model, with the host proteins being known as ‘guardees’. A sophisticated advancement of the ‘guard’ model involves ‘decoy’ proteins. In contrast to the guardees, decoy proteins do not have a function in plant immunity; their sole role is to mimic effector targets and to trap virulence effectors. If a decoy is monitored by an NLR receptor, effector binding to the decoy will trigger a defense response (Fig. 1).

## Some NLRs are chimeric and have integrated decoy domains

Interestingly, decoys and the decoy-monitoring NLR proteins are sometimes encoded by the same gene; these are known as ‘integrated domain’ NLRs (NLR-IDs). Two independent studies recently described the functional analysis of the *Arabidopsis* NLR protein RRS1, which contains an additional WRKY domain [4, 5]. WRKY domains are part of transcription factors that play important roles in signal transduction during basal plant immunity, and consequently are often targets of effector proteins. Indeed, the WRKY domain of RRS1 was shown to be targeted by two effector proteins of different pathogens and effector binding led to the activation of RRS1. It was concluded that the WRKY domain of RRS1 represents an inbuilt decoy that traps effectors and activates plant defense. Similarly, the NLR proteins RGA5 and Pik-1 of rice contain a heavy metal-associated (HMA) domain that is targeted by several effectors of the rice blast pathogen *Magnaporthe oryzae* [6, 7]. These two examples provided the first experimental studies of NLR-IDs.

In their *BMC Biology* article, Sarris et al. [1] made use of whole-genome sequence information from plants and undertook a systematic search for NLR-IDs in different plant species, ranging from mosses to cereals and trees. Interestingly, putative NLR-IDs are frequently and commonly found in almost all plant species, though their number varies considerably among species. For example, only one NLR-ID was found in cucumber, whereas 93 were identified in apple. Ultimately, the study identified 720 NLR-IDs in 37 plant species. The authors also found that although the number of NLRs varies across species of flowering plants, about 10 % of all NLRs contained an integrated domain. In total, 265 distinct integrated domains were identified — a massive increase from the 22 NLR-IDs previously described [8]. Of these, 61 occurred in more than one plant family, with the protein kinase domain being the most frequent. Kinase domain fusions were sometimes found with an additional fused domain that increases the structural diversity of such proteins.

Some of the domain fusions have been conserved during evolution, but many probably evolved through convergent evolution, resulting in similar NLR-IDs across different plant lineages. For example, there were at least five independent events in which WRKY domains were fused to NLRs in different plant families. The observation that recruitment of certain domains into NLRs occurred independently in different plant families suggests that these fusions have a selective advantage for the plant. The fusion of integrated domains and NLRs ensures that the decoy and the corresponding immune receptor are inherited together; separate coding of the decoy and NLR bears the risk that the two genes are separated during meiosis, resulting in susceptible offspring. In addition, integrating the decoy domain into the respective NLR reduces the risk of hybrid incompatibility between different alleles of NLRs and decoys.

Since some integrated domains (such as the protein kinase, WRKY or RIN4 domains described above) have previously been shown to be targets of effector proteins, the newly identified integrated domains represent new candidate effector targets. The study identified several integrated domains that have not yet been reported as effector targets. These protein domains most probably play an as yet unidentified role in plant immunity.

## Are there more chimeric NLRs to be identified?

The study of Sarris et al. [1] identifies many previously unknown NLR domain fusions. Nevertheless, it is likely that an even greater diversity of these domain fusions is encoded in the genomes of higher plants. Many plant genome assemblies are still of relatively poor quality in terms of both fragmentation and annotation, which hampers the correct identification of NLR-IDs. The quality of assemblies will increase in the near future thanks to recent improvements in sequencing and assembly technologies. Furthermore, the current identification of NLR-IDs is based on clearly identifiable integrated domains. We hypothesize that smaller domains representing the remnants of a complete decoy protein domain could be present in some NLR-IDs. Such domains might have been reduced by deletion events, without loss of the decoy function. The sometimes rather ill-defined C-terminal regions of LRR domains or ‘spacer’ domains between the individual subdomains of NLRs might actually represent candidates for such cryptic decoy domains. They might consist of only short peptide regions in the form of epitopes, and it seems that such regions are worthy of closer examination.

Another interesting aspect and perspective of the Sarris et al. article relates to the observation that NLR-IDs seem to work in pairs of closely linked NLRs, at least in the cases studied in detail to date [8]. Thus, we expect that for every new NLR-ID, a partner NLR is also involved in resistance interaction.

## New leads for research and breeding

As fusion domains have been shown to act as targets for pathogen effectors, each newly identified integrated domain provides a promising lead for studying how pathogens manipulate host metabolism. Thus, the work by Sarris et al. represents a rich resource for new research projects. Initial experiments might aim to identify effectors that target these newly identified domains. An important question pertaining to all resistance mechanisms relates to their durability in a crop cultivar grown on a large area and over several years. NLR-mediated immunity is often not durable because effector modification can disrupt recognition by the respective immune receptor, resulting in the emergence of new, virulent pathogen races. Because pathogens can modify their effector repertoire very rapidly, NLR genes are frequently overcome in the field, sometimes only a few years after the release of a new crop cultivar. There is no evidence that NLR-IDs provide a more durable resistance than normal NLRs. For example, the Lr10–RGA2 pair, in which an additional nucleotide-binding domain is integrated in RGA2, does not provide durable resistance; virulent races that overcame Lr10–RGA2 resistance are commonly found [8, 9]. Similarly, nonsynonymous polymorphisms and deletions in the rice blast effector *Avr-Pia* result in virulence to RGA5, indicating that these modifications in *Avr-Pia* disrupt recognition by RGA5 [6]. Nevertheless, more durable resistance might be achieved with knowledge of integrated domains: very interestingly, the rice Pi21 protein, like RGA5 and Pik-1, also contains a HMA domain [7]. Loss of *Pi21* results in durable resistance [10], indicating that the *Pi21* gene is a susceptibility factor. This suggests that the HMA domain is a target of a pathogen effector. Therefore, integrated domains could provide information about susceptibility factors that might serve as targets to generate knock-out mutations in the host that have the potential to provide durable resistance. Novel gene-editing technologies in crops provide the tools necessary to achieve this relatively easily.

## Abbreviations

HMA: Heavy metal-associated
NLR: Nucleotide-binding, leucine-rich repeat proteins
NLR-ID: NLR integrated domain
